# Effects of Antidepressant Treatment on Peripheral Biomarkers in Patients with Major Depressive Disorder (MDD)

**DOI:** 10.3390/jcm10081706

**Published:** 2021-04-15

**Authors:** Anna Mosiołek, Aleksandra Pięta, Sławomir Jakima, Natalia Zborowska, Jadwiga Mosiołek, Agata Szulc

**Affiliations:** 1 Department of Psychiatry, Faculty of Health Sciences, Medical University of Warsaw, Żwirki i Wigury 61 Street, 02-091 Warszawa, Poland; aleksandrapieta123@gmail.com (A.P.); agataszulc@poczta.onet.pl (A.S.); 2 Mazovia Specialist Health Center in Pruszków, Partyzantów 2/4 Street, 05-802 Pruszków, Poland; s.jakima@wp.pl (S.J.); nataliazborowska8@gmail.com (N.Z.); 3 Faculty of Medicine, Wroclaw Medical University, Wybrzeże Ludwika Pasteura 1 Street, 50-367 Wrocław, Poland; jadmosiolek@gmail.com

**Keywords:** depression, inflammatory cytokines, response to antidepressants

## Abstract

Major depressive disorder (MDD) is one of the most prevalent mental illness and a leading cause of disability worldwide. Despite a range of effective treatments, more than 30% of patients do not achieve remission as a result of conventional therapy. In these circumstances the identification of novel drug targets and pathogenic factors becomes essential for selecting more efficacious and personalized treatment. Increasing evidence has implicated the role of inflammation in the pathophysiology of depression, revealing potential new pathways and treatment options. Moreover, convergent evidence indicates that MDD is related to disturbed neurogenesis and suggests a possible role of neurotrophic factors in recovery of function in patients. Although the influence of antidepressants on inflammatory cytokines balance was widely reported in various studies, the exact correlation between drugs used and specific cytokines and neurotrophins serum levels often remains inconsistent. Available data suggest anti-inflammatory properties of selective serotonin reuptake inhibitors (SSRIs), selective serotonin and noradrenaline inhibitors (SNRIs), and tricyclic antidepressants (TCAs) as a possible additional mechanism of reduction of depressive symptoms. In this review, we outline emerging data regarding the influence of different antidepressant drugs on a wide array of peripheral biomarkers such as interleukin (IL)-1ß, IL-2, IL-5, IL-6, IL-8, IL-10, C-reactive protein (CRP), or interferon (IFN)-γ. Presented results indicate anti-inflammatory effect for selected drugs or lack of such effect. Research in this field is insufficient to define the role of inflammatory markers as a predictor of treatment response in MDD.

## 1. Introduction

Depression is the most commonly diagnosed psychiatric disorder. It has a multifactorial etiology and the various hypotheses of depression are complementary to one another. Major depression has been associated with symptoms of immune activation, the phenomenon of oxidative stress, changes in immune processes, and activation of the inflammatory response system (IRS) [1,2,3,4,5,6,7,8,9,10,11,12,13,14,15]. Recognized markers of inflammation include: inflammatory enzymes, pro-inflammatory cytokines, and anti-inflammatory cytokines.

Studies suggest that depression is associated with elevated levels of both pro-inflammatory and anti-inflammatory cytokines, e.g., IL-1β, IL-6, IFN-γ, TNF-α, and C-reactive protein (CRP). Cytokines are a diverse group of biochemical compounds produced by immunocompetent cells of the immune system: lymphocytes, macrophages, and natural killer (NK) cells. The inflammatory hypothesis of depression, also referred to as the cytokine hypothesis, suggests that pro-inflammatory cytokines, acting as neuromodulators, affect the neurochemical, behavioral, and neuroendocrine features of depression [3,4,13,14,15]. Pro-inflammatory cytokines may cause hypothalamic–pituitary–adrenal (HPA) axis hyperactivity by interfering with the negative feedback of the HPA axis and corticosteroids (CS) and may reduce serotonin (5-HT) levels by decreasing the availability of its precursor, tryptophan (TRP), through the activation of a TRP-metabolizing enzyme, indoleamine 2,3-dioxygenase (IDO) [3,4,8,14,15,16,17]. Excessive HPA-axis activity and immune dysregulation also lead to abnormal function of the kynurenine pathway responsible for tryptophan conversion to two key compounds involved in mood regulation: serotonin and melatonin [16]. According to the kynurenine hypothesis of depression, inflammatory factors cause excessive activation of IDO, an enzyme found in microglia, astrocytes, and neurons [14,15,16]. Depression is therefore associated with neuro–immuno–metabolic interactions and immunometabolic dysregulation.

Aim: This clinical review aimed to summarize available research and describe the correlation between selected antidepressants and peripheral inflammatory biomarkers in patients diagnosed with major depressive disorder (MDD).

## 2. Materials and Methods

The PubMed and Web of Science databases were systematically searched for human studies published in peer-reviewed journals from January 1, 2000, to November 11, 2020. The selection criteria for publications to be included in the review were as follows: clinical diagnosis of MDD (without comorbidities); study duration of at least 6 weeks; assessment of inflammatory markers during treatment with any of the following drugs: sertraline, paroxetine, fluoxetine, escitalopram, mirtazapine, mianserin, tianeptine, ketamine, vortioxetine, duloxetine, venlafaxine, trazodone; at least 2 measurements of the marker during the study (at baseline and at the end of the study); study population of more than 20 subjects; subject age range of 18–65 years. Exclusion criteria were as follows: group of subjects <20, measurements taken at one time point only, duration <6 weeks, specific population (adolescents, menopausal women, other severe comorbidities).

After following the inclusion and exclusion criteria, there were no sufficient or quality data for mirtazapine, mianserin, tianeptine, ketamine, vortioxetine, and trazodone. The search terms included: depression, cytokines, antidepressant drug, and their synonyms. The following factors were included in the review: IL-1, IL-1β, IL-2, IL-4, IL-5, IL-6, IL-8, IL-10, IL-12, CRP, TNF-α, IFN-γ. The databases were searched separately for each factor according to the search criteria described above. The data were searched and extracted separately by four authors, the results were compared, and consensus was reached through discussion.

## 3. Results

### 3.1. Inflammatory Cytokines 

Studies confirm that depressive disorders, in the absence of other physical comorbidities, are associated with increased levels of various pro-inflammatory cytokines, including tumor necrosis factor alpha (TNF-α) and interleukins (ILs). According to the cytokine hypothesis of depression proposed by Maes, markers of inflammation play the key role in the development of depressive symptoms [1,2]. Cytokines are most commonly classified according to their effects on inflammation into cytokines that stimulate the development of inflammation, i.e., pro-inflammatory cytokines (e.g., interferon (IFN)-γ, TNF, IL-1, IL-2, IL-5, IL-8) and those that suppress inflammation, i.e., anti-inflammatory cytokines (e.g., IL-1β, IL-6, TNF-α, IL-10, IL-19, IL-20, IL-22, IL-24, IL-26, IL-28, IL-29). There is also a group of cytokines categorized as either anti- or pro-inflammatory, depending on the circumstances (e.g., IL-6, TGF-β, INF-α).

### 3.2. Interferon Gamma (IFN-γ)

A total of 171 records were displayed using various combinations of the following search terms: IFN-γ, antidepressants, SSRI (selective serotonin reuptake inhibitor), SNRI (selective serotonin and noradrenaline inhibitor), venlafaxine, sertraline, escitalopram, citalopram, mianserin, trazodone, agomelatine, duloxetine. Relevant records numbered 27. The search criteria were met by six studies and three meta-analyses.

Interferon gamma (IFN-γ) is a pro-inflammatory cytokine produced by immune cells, principally Th1 cells, cytotoxic lymphocytes, B cells, and antigen-presenting cells. IFN-γ induces indoleamine 2,3-dioxygenase (IDO), which catalyzes conversion of tryptophan to kynurenine in the kynurenine pathway. This reaction results in decreased serotonin levels and overactivity of the glutamatergic system, which have been linked to depressive symptoms [3,4,13,14,15,16,18]. Given the evidence suggesting the potential involvement of IFN-γ in the pathogenesis of MDD, this cytokine has been investigated in numerous studies. The present review included studies that assessed the effects of treatment of MDD patients with venlafaxine, paroxetine, duloxetine, and sertraline on IFN-γ.The results concerning the effect of these antidepressant drugs on IFN-γ levels obtained by the respective research teams and presented in this review are conflicting.

In the study by Chen et al. [19], patients with MDD underwent 8 weeks of treatment with venlafaxine (75–300 mg) or paroxetine (10–40 mg). The dose of the drug was individually adjusted to the severity of symptoms. Baseline IFN-γ levels (W0) were significantly higher in the group of patients (*n* = 91) compared with controls (*n* = 90) (*p* < 0.001). In the group treated with venlafaxine (*n* = 41), decreased IFN-γ levels were observed after 8 weeks of treatment (W8) (*p* < 0.001). The reduction in IFN-γ levels was statistically non-significant in remitters (*n* = 15) (*p* = 0.136) and significant in non-remitters (*p* = 0.002). There were no significant differences in IFN-γ levels between subjects who had responded to venlafaxine treatment and those without remission (*p* = 0.667 at W0, *p* = 0.978 at W8). After 8 weeks of treatment, IFN-γ continued to be significantly higher compared with controls (*p* < 0.001 for remitters and *p* < 0.001 for non-remitters). In the group treated with paroxetine (*n* = 50), IFN-γ levels increased after 8 weeks of treatment (*p* = 0.047). At W0, IFN-γ levels were significantly higher than those in the group of healthy individuals (*p* < 0.001). IFN-γ levels increased among patients with remission (*p* = 0.456 for W0 vs. W8), and the IFN-γ increase after 8 weeks was statistically significant (*p* = 0.014). While the difference in IFN-γ levels between the remitters and non-remitters at baseline (W0) was non-significant (*p* = 0.891), the IFN-γ levels in non-remitters after 8 weeks of treatment were significantly higher (*p* = 0.026). At the end of the study (Week 8), IFN-γ continued to be significantly higher compared with controls (*p* < 0.001 for remitters and *p* < 0.001 for non-remitters). After 8 weeks of treatment, the mean IFN-γ level in the venlafaxine group was significantly lower than that in the paroxetine group (*p* < 0.001). IFN-γ levels decreased after treatment with venlafaxine (*p* < 0.001) and increased after treatment with paroxetine (*p* = 0.047). These findings therefore suggest differential effects of these drugs on inflammatory factors. Studies in a larger sample of patients are needed [19].

Similarly, Dahl et al. reported in their study significantly elevated IFN-γ levels in untreated patients with depression (*n* = 50) compared with healthy individuals (*p* = 0.043). The selection of antidepressant drug in this group was based on clinical assessment, and the effects of individual agents were not investigated in the study. SSRIs, SNRIs, mirtazapine, benzodiazepines, lamotrigine, and psychotherapy were used. Patients who completed their treatment had their cytokine levels measured after 12 weeks, and a decrease in IFN-γ from baseline was observed (*p* = 0.036). In the group of patients who achieved remission (*n* = 30), IFN-γ levels were significantly decreased compared with baseline (*p* = 0.043) [20].

A study (Hernandez et al., 2013) conducted in a small group of patients treated with SSRIs (*n* = 31) demonstrated that IFN-γ levels fluctuated during the treatment and significantly varied (*p* < 0.0001) depending on the time point at which they were measured after commencement of the treatment. At Week 5 (W5), IFN-γ levels were significantly higher compared with those on the day of inclusion (*p* < 0.0001). In subsequent weeks, a decrease in IFN-γ levels was observed, while the levels of IFN-γ at Week 52 (W52) were comparable to those in the control group [21]. In a 6-week study (Brunoni et al., 2014) (*n* = 73) in patients treated with sertraline alone, IFN-γ levels decreased after 6 weeks (W6), with the decrease being independent of the treatment response. In this study, baseline plasma levels of the cytokines, including IFN-γ, did not predict the response after 6 weeks of treatment [22].

In a study by Fornaro et al. (2013), levels of cytokines, including IFN-γ, were monitored at baseline and at 6 and 12 weeks of treatment with duloxetine at the dose of 60 mg/day in patients with MDD (*n* = 30) and in controls (*n* = 32). The patients were divided in two groups depending on treatment response: early responders (ER), defined as those with >50% reduction in their Hamilton Depression Rating Scale (HAM-D) score from baseline to Week 6 (T6) and early non-responders (ENR). After 6 weeks of duloxetine treatment, IFN-γ levels significantly increased in the ENR group (*p* = 0.021). Compared with the control group, early responders and early non-responders showed lower levels of IFN-γ throughout the study (*p* = 0.000). No significant differences in cytokine levels were observed between ER and ENR. At Week 12 (W12), no statistically significant changes in plasma IFN-γ levels were observed in ENR. No significant differences in the absolute values of cytokine levels at Week 6 were reported between patients who demonstrated clinical response and non-responders [23]. The above findings are presented in Table 1.

The meta-analyses concerning the effects of SSRIs, SNRIs, and TCAs also failed to demonstrate any significant effect of the treatment on IFN-γ levels in patients with MDD (*p* > 0.05) [24,25,26]. Most of the studies showed that IFN-γ levels were lower in patients with MDD before the initiation of treatment with antidepressant drugs [22,23], and these reports have been confirmed by a meta-analysis that compared pre-treatment results in MDD patients (*n* = 154) with those in healthy individuals [25]. Chen et al. (2017) and Dahl et al. (2014) reported significantly increased levels of IFN-γ in untreated patients with depression [19,20]. This review shows that the results concerning IFN-γ levels before the treatment of MDD and after its initiation are equivocal and require further studies. Studies have shown increased INF-γ levels after treatment with duloxetine and paroxetine [19,23] and decreased IFN-γ levels after treatment with venlafaxine and sertraline [19,22]. Baseline IFN-γ levels in patients with MDD did not predict the response after 6 weeks of treatment [22].

### 3.3. Interleukin-1 (IL-1)

A total of 211 records were displayed using various combinations of the following search terms: IL-1, interleukin 1, antidepressants, SSRI, SNRI, venlafaxine, sertraline, escitalopram, citalopram, mianserin, trazodone, agomelatine, duloxetine. Relevant records numbered 28. The records that met the selection criteria included seven studies and one meta-analysis.

A significant role in the pathogenesis of depressive states is attributed to interleukin-1 (IL-1), which is important for the regulation of multiple brain processes, including sleep and food intake, which are disturbed in depression. IL-1 plays the role of a universal factor that stimulates inflammation and is produced in response to various types of antigens. It is also capable of stimulating cells to produce other pro-inflammatory cytokines [37]. The term “interleukin-1” refers to a whole group of cytokines that play a key role in initiating inflammation. Because of the similar biological activity and the same receptor involved in signal transduction, IL-1α (IL-1F1) and IL-1β (IL-1F2) are often described as just one IL-1. They do, however, differ, for instance, in terms of the cells in which they are synthesized. IL-1α is synthesized by monocytes, macrophages, neutrophils, lymphocytes, glial cells, keratinocytes, and endothelial cells, while IL-1β is mainly synthesized by monocytes/macrophages. Another difference is that IL-1β is synthesized in the form of a precursor (pro-interleukin-1β) that is converted to the mature form by caspase-1. IL-1β is secreted to blood and acts systemically. This cytokine also affects the activity of the HPA axis by means of a negative feedback relationship between IL-1β production and overactivity of the HPA axis, whose dysregulation may play a role in initiating and maintaining the clinical and biochemical manifestations of depression [38,39]. A total of six clinical studies were included in the analysis of the effects of antidepressant drugs on IL-1 levels.

In these studies, IL-1β levels were significantly higher at baseline in patients with MDD than those in healthy individuals [19,23,29,40,41]. Chen et al. assessed the effect of venlafaxine (*n* = 41) and paroxetine (*n* = 50) on IL-1 levels in patients diagnosed with MDD [19]. After 8 weeks of treatment (W8) with venlafaxine or paroxetine, the mean IL-1β levels decreased in both treatment groups (*p* = 0.024) and were eventually similar to those in healthy individuals (*p* < 0.001). The study also showed that IL-1 levels during treatment with venlafaxine were lower than those during paroxetine treatment [19]. Piletz et al. (2009), however, reported quite opposite results [40]. While the baseline levels of IL-1β in patients with MDD were higher than those in healthy individuals (*p* = 0.03), the levels of this cytokine after 8 weeks of treatment with venlafaxine (W8)—in contrast to the study by Chen et al. [19]—increased [40]. In a study by Pérez-Sánchez et al. (2018), the Mann–Whitney U test revealed significant differences in the levels of IL-1β (*Z* = 2.8409; *p* < 0.01). During the fourth week of treatment with fluoxetine, patients with MDD had significantly higher IL-1β levels than did healthy individuals, while after 8 weeks of treatment, IL-1β returned to baseline (*p* < 0.005). ANOVA showed that fluoxetine was a significant predictor of the change in IL-1β levels (*F* = 15.58; *p* < 0.0001) [41]. Similar results concerning pre-and post-treatment IL-1β levels have also been reported by Hernandez et al. After treating MDD with fluoxetine, paroxetine, and sertraline, IL-1β levels were comparable with those in healthy individuals [21]. Fornaro et al. (2013) identified in their study a group of subjects who responded to duloxetine early (within 6 weeks) and compared them with the group of patients who did not achieve such a rapid response and with the group of healthy individuals. They observed significant differences in IL-1β levels (*F* = 1.827, df = 59.6; *p* = 0.000) compared with the study group and the control group. Patients achieving clinical improvement after 6 weeks of treatment with duloxetine had higher IL-1β levels compared with those who did not improve with the treatment (*F* = 3.226, df = 15.7; *p* = 0.032). The levels of IL-1β in patients with a later response to the drug were lower than those in healthy individuals but subsequently increased after 12 weeks of treatment [23]. A lack of significant differences in the levels of IL-1β before and after treatment with fluoxetine has been reported by Jazayeri et al. [29]. The review of studies assessing the effects of antidepressant drugs on IL-1 levels shows that the results for IL-1 levels after initiation of treatment with those drugs are equivocal and require further studies. A significant increase in the secretion of IL-1β has been demonstrated in patients with depression. Varying levels of IL-1 have been reported depending on the drug used and the timing of measurements. There have been reports of no effect of antidepressant drugs on IL-1 levels [29,30], reports of decreased levels [19], and reports of increased levels [21,23,40] after treatment with antidepressant drugs. Patients achieving clinical improvement had higher IL-1β levels compared with those who had not achieved improvement with the treatment, and patients with a later response to the drug had lower IL-1β levels compared with those in healthy individuals [23].

### 3.4. Interleukin-6 (IL-6)

A total of 437 records were displayed using various combinations of the following search terms: IL-6, interleukin 6, antidepressants, SSRI, SNRI, venlafaxine, sertraline, escitalopram, citalopram, mianserin, trazodone, agomelatine, duloxetine. Relevant records numbered 34. The records that met the selection criteria included seven studies and one meta-analysis.

Pro-inflammatory properties of IL-6 and its elevated levels in patients with depression were reported in most studies. This is usually accompanied by elevated levels of acute phase proteins. It has even been proposed that IL-6 elevation should be considered a biological marker of depression [42].

Carboni et al. (2019) compared IL-6 levels in the control group and in the group of patients with MDD treated with paroxetine (*n* = 52) and venlafaxine (*n* = 51) [27]. IL-6 levels were measured at baseline (W0) and after 10 weeks (W10). In patients treated with paroxetine, IL-6 positively correlated with the HAM-D score after 10 weeks of treatment (*r* = −0.23, *p* = 0.016), where the negative signs indicate a correlation between biomarker increase and HAMD score reduction. These correlations were stronger in women. No significant correlations were, however, observed in the venlafaxine group [27]. In a study by Pérez-Sánchez et al. (2018), baseline IL-6 levels in patients were higher than those in healthy individuals. The Mann–Whitney U test revealed significant differences in IL-6 levels (*Z* = 2.86816; *p* < 0.01) between the two groups. During the fourth week of treatment with fluoxetine, patients with MDD had significantly lower IL-6 levels compared with the levels on the day of inclusion in the study, while after 8 weeks of treatment (W8), IL-6 returned to baseline (*p* < 0.05). Analysis of variance showed a significant change in the treatment with fluoxetine for IL-6 (*F* = 3.354; *p* < 0.05, df = 2/65) [41]. The IL-6 levels observed in the study depended on the timing of antidepressant intake. Similarly, in a study by Manoharanet et al. (2016), IL-6 levels were higher in patients compared with healthy individuals (*p* < 0.01). Patients who responded to fluoxetine treatment (39 responders) did not differ in terms of pre-treatment IL-6 levels from those who did not respond to the treatment (34 non-responders) (*p* = 0.58). Additionally, no significant reduction in IL-6 levels was observed in either group after treatment with fluoxetine (*p* = 0.05 for responders, *p* = 0.34 for non-responders). A significant correlation was, however, observed between the percentage change in IL-6 levels and the HAM-D score in responders (Pearson *r* = 0.38; *p* = 0.0173) [28]. In a study by Yoshimura et al. (2013), IL-6 levels were significantly higher in patients responding to the study drugs, paroxetine (*n* = 66) and sertraline (*n* = 42), compared with non-responders (*p* = 0.0328). Moreover, the baseline, pre-treatment levels of IL-6 positively correlated with the results of a questionnaire measuring the clinical symptoms of depression (*r* = 0.167, *p* = 0.07014). In the respondents, IL-6 levels significantly decreased after 8 weeks of treatment (*p* = 0.029), while no such changes were observed in patients with no improvement in the clinical symptoms of depression. A significant correlation was also observed between the changes in HAM-D scores and the changes in IL-6 levels (*r* = 0.246, *p* = 0.007) [43]. The review of the studies assessing the effects of antidepressant drugs on IL-6 levels revealed considerable discrepancies in study results. In all the studies included in the review, IL-6 levels in patients with MDD were significantly higher than those in healthy individuals [27,28,41,43], but the effects of the study drugs on IL-6 levels varied. These effects also varied depending on the timing of the second measurement of the IL-6 level. In a study by Manoharanet al. (2016), patients who favorably responded to the treatment did not differ in terms of pre-treatment IL-6 levels from non-responders [28], while in the study by Yoshimura et al. (2013), IL-6 levels were significantly higher in patients favorably responding to the drugs [43]. In patients responding to the treatment, IL-6 levels significantly decreased after treatment [27,41,43].

### 3.5. Interleukin-8 (IL-8)

A total of 85 records were displayed using various combinations of the following search terms: IL-8, interleukin 8, antidepressants, SSRI, SNRI, venlafaxine, sertraline, escitalopram, citalopram, mianserin, trazodone, agomelatine, duloxetine. Relevant records numbered four. The records that met the selection criteria included three studies and one meta-analysis.

Interleukin-8 is not only a pro-inflammatory cytokine but also a chemotactic factor that can stimulate migration of immune cells in the body, thereby affecting the immune response. This means that it stimulates migration and propagation of T cells, neutrophils, and monocytes. This is a protective effect. IL-8 stimulates histamine release from basophils, which results in an allergic reaction. IL-8 is released by monocytes, endothelial cells, and skeletal muscle cells in response to increasing concentrations of IL-1β, TNF-α, and reactive oxygen species.

Chen et al. assessed the effect of venlafaxine (*n* = −41) and paroxetine (*n* = −50) on interleukin levels in patients diagnosed with MDD. In this study, IL-8 levels were higher in patients compared with healthy individuals (*p* < 0.001). After 8 weeks of treatment, the mean IL-8 levels decreased and did not differ significantly from those in healthy individuals (*p* = 0.491). The decrease was greater in patients treated with venlafaxine than those treated with paroxetine (*F* = 18.539, df = 1.78, *p* < 0.001) [19]. Eller et al. (2008) assessed the effect of escitalopram on IL-8 levels in a group of patients with MDD (*n* = 100) versus controls (*n* = 42). Seventy-four patients responded to escitalopram treatment. However, no significant difference was observed in the mean IL-8 levels between the responders and non-responders (*p* > 0.05). The duration of drug effect was also irrelevant (*F* = 2.18, df = 2, *p* = 0.118). Cytokine levels were measured at baseline and after 4 and 12 weeks of the experiment. No correlations between IL-8 and depression markers were observed [31]. Levels of IL-8 were either unaffected [30,31] or decreased [19,31] following antidepressant treatment with venlafaxine and sertraline. Two studies confirm the lack of effect on IL-8 levels by escitalopram [30,31].

### 3.6. Tumour Necrosis Factor (TNF)

A total of 403 records were displayed using various combinations of the following search terms: TNF, antidepressants, SSRI, SNRI, venlafaxine, sertraline, escitalopram, citalopram, mianserin, trazodone, agomelatine, duloxetine. Relevant records numbered 35. The records that met the selection criteria included six studies and one meta-analysis.

Another mediator of inflammation that plays a significant role in the pathogenesis of depression is TNF-α, which is produced in small amounts by neurons and microglia. Elevation in plasma TNF levels is believed to play the role of a mediator in patients with depression compared with healthy individuals [44] (Almond, 2013). Most studies have reported elevated TNF levels in patients with MDD compared with healthy individuals [27,31,41,44]. However, Fornaro et al. (2013) did not observe any differences in pre-treatment TNF levels between healthy individuals (*n* = 32) and patients with MDD (*n* = 30) [23]. Eller et al. (2008) evaluated in their study 100 patients with MDD and 42 healthy individuals. Seventy-four patients showed a good response to escitalopram. When the mean TNF levels were compared, they were found to differ significantly between these groups. Patients with a favorable response to the drug had lower TNF levels compared with controls (*p* < 0.05) and with non-responders, while the controls and non-responders did not differ significantly between one another in terms of TNF levels [31]. No significant differences were, however, demonstrated between TNF levels before and after treatment with escitalopram. There was also no correlation with the results obtained in the questionnaire quantifying clinical symptoms of depression, the Montgomery-Asberg Depression Rating Scale (MADRS) [31].

Similar results were obtained by Halaris et al. (2015). Their study demonstrated that TNF levels changed over the course of escitalopram treatment (3.33 at W0, 1.92 at W8, 2.35 at W12; *p* = 0.07), although the changes were not statistically significant (*p* > 0.05) [30]. Carboni et al. (2019) observed in a group of patients treated with paroxetine a significant correlation between TNF elevation and HAM-D questionnaire scales (*r* = −0.22, *p* = 0.020) after 10 weeks of treatment (W10). They also found that higher baseline levels of TNF correlated with better treatment responses. Such correlations were not, however, observed in the group of patients treated with venlafaxine (*p* > 0.05) [27]. Pérez-Sánchez et al. (2018) demonstrated significant changes in TNF levels during the treatment with fluoxetine (*F* = 5.074; *p* < 0.05, df = 2/65). The Mann–Whitney U test revealed significant differences in TNF-α levels (*Z* = 3.23518; *p* < 0.01). During the fourth week of treatment with fluoxetine, patients with MDD had significantly lower TNF levels, but after 8 weeks of treatment, TNF levels returned to baseline (*p* < 0.05) [41]. Fornaro et al. (2013) identified in their study a group of subjects who responded to duloxetine early (within 6 weeks) and compared them with the group of patients who did not achieve such a rapid response and with the group of healthy individuals. They noticed significant differences in TNF levels between the group of responders and the group of non-responders (*F* = 3.812, df = 47.9; *p* = 0.022). The patients achieving clinical improvement after 6 weeks of treatment with duloxetine had lower TNF levels compared with those who did not improve with the treatment (*p* = 0.000) [23]. The above findings are summarized in Table 1.

As shown in Table 1, the effects of antidepressant drugs on TNF vary. There have been reports of no effect of antidepressant drugs on TNF levels [19,27,30,31], reports of increased TNF levels [23,27,41], and reports of decreased levels of this cytokine [19]. While escitalopram treatment did not affect TNF levels [30,31], data on the effects of venlafaxine, paroxetine, fluoxetine and duloxetine are equivocal [19,23,27,41]. Higher baseline TNF levels correlated with better treatment responses.

### 3.7. Interleukin-10 (IL-10)

A total of 142 records were displayed using various combinations of the following search terms: IL-10, interleukin 10, antidepressants, SSRI, SNRI, venlafaxine, sertraline, escitalopram, citalopram, mianserin, trazodone, agomelatine, duloxetine. Relevant records numbered 11. The records that met the selection criteria included five studies and one meta-analysis.

The last interleukin included in the present review is the anti-inflammatory interleukin IL-10. Its main task is to suppress inflammation. It is produced by B cells, macrophages, and dendritic cells. It minimizes the effects of inflammation on physical exertion by suppressing the production of pro-inflammatory cytokines TNF-α and IL-8 and stimulates the synthesis of IL-1 receptor antagonist in muscles. Data on IL-10 levels in patients with MDD are equivocal. The study by Fornaro et al. (2013) cited above did not demonstrate any significant differences in IL-10 between patients and healthy individuals at baseline and at the end of the study. IL-10 levels initially decreased after 6 weeks of treatment with duloxetine (*p* = 0.041) and then returned to baseline after 12 weeks of treatment [23]. Carboni et al. compared two groups in their study: a group consisting of patients with MDD treated with paroxetine (*n* = 52) and controls (*n* = 54) and a group that comprised patients with MDD treated with venlafaxine (*n* = 51) and controls (*n* = 53). Cytokine levels were measured before treatment initiation and after 10 weeks of treatment (W10). Before the experiment, the group of patients treated with paroxetine displayed a correlation between IL-10 and HAM-D scores (*r* = 0.19, *p* = 0.045). Such correlations were not, however, observed in the group of patients treated with venlafaxine (*p* > 0.05). After 10 weeks of treatment with paroxetine, IL-10 levels positively correlated with HAM-D scores (*r* = −0.23, *p* = 0.022)), where the negative signs indicate a correlation between biomarker increase and HAMD score reduction. Higher baseline IL-10 levels correlated with better treatment responses. These correlations were stronger in men. It was also found that the baseline IL-10 levels differ significantly (*p* = 0.0099) in patients who favorably responded to paroxetine (*n* = 36) compared with the patients who did not achieve improvement with the treatment. No such effect was observed in the placebo group. No significant correlations were, however, observed in the venlafaxine group [27]. Most studies failed to confirm any effect of antidepressant drugs on IL-10 levels [19,30,41]. Carboni et al. (2019) observed an effect of paroxetine on IL-10 levels, which might mean that IL-10 could be a significant predictor of response to paroxetine treatment [27]. Higher baseline IL-10 levels correlated with better treatment responses.

### 3.8. C-Reactive Protein (CRP)

A total of 91 records were displayed using various combinations of the following search terms: CRP, major depressive disorder, SSRI, SNRI, ketamine, antidepressants, sertraline, escitalopram, citalopram, mianserin, trazodone, agomelatine, venlafaxine, duloxetine. Relevant records numbered 36. The records that met the selection criteria included four studies, one meta-analysis, and one review (Table 1).

C-Reactive protein is a non-specific acute phase protein whose levels increase in response to systemic inflammation. CRP is used in psychiatry as a marker of chronic inflammation [45]. Meta-analyses of cross-sectional studies confirm that the mean concentrations of circulating CRP and inflammatory cytokines are higher in patients with a depressive episode compared with controls [46,47]. These findings formed the basis for studies investigating the use of CRP as a prognostic factor for the response to antidepressant treatment [48]. In cases of treatment with fluoxetine (*n* = 73) and venlafaxine (*n* = 76), the baseline CRP levels significantly correlated with treatment response at 2 weeks (*p* = 0.02). Higher CRP values were associated with a lower treatment efficacy. After 6 weeks, CRP significantly increased with both treatments (*p* < 0.001) [32]. Paroxetine treatment in patients with MDD (*n* = 52) elevated CRP levels, which was correlated with a reduction in depressive symptoms on the HAM-D scale (*p* < 0.001) [27]. Baseline CRP levels were associated with subsequent responses to the drug. Early responders (those responding after 6 weeks) showed higher CRP levels (*p* = 0.008) than non-responders. Late responders (those responding after 12 weeks) also displayed moderately elevated CRP levels, although to a statistically significant degree (*p* = 0.054) [49]. A comparison of CRP levels in a group of patients with MDD (*n* = 26) treated with SSRIs for 6 weeks revealed that CRP levels at the end of the study were significantly lower than those observed on admission (*p* = 0.011) and decreased to values comparable with those in the control group (*p* = 0.01) [36]. A meta-analysis of nine studies (*n* = 325) assessing the effects of antidepressant drugs (SSRIs, SNRIs, TCAs) on CRP levels did not show any statistically significant effect of the treatments on CRP (*p* > 0.05) [25]. Data on the effects of these drugs on CRP are equivocal. The meta-analysis by Więdłocha et al. (2018) suggests no effect of these drugs, and the effects in the studies included in this review vary [25]. The effects of antidepressants on various cytokines are summarized and presented in Table 2.

## 4. Discussion

Studies show that antidepressant treatment based on monoamine neurotransmitter regulation has certain limitations. There is a large group of patients who fail to achieve treatment response or whose response is unsatisfactory. Developing a treatment model for depression that would be based on brain neurochemistry seems to be a more personalized and therefore more effective therapeutic strategy. Studies have demonstrated an association between immune dysregulation, inflammation, and the initiation and exacerbation of major depressive disorder (MDD) [1,2,3,4,5,6,7,8,9,10,11,12,13,14,15,18]. The inflammatory hypothesis of depression suggests that cytokines play a key role in the pathophysiology of MDD, and changes in peripheral levels of cytokines have been linked to the outcomes of antidepressant treatment. Predicting the response to antidepressant drugs poses a challenge in MDD. Selective serotonin reuptake inhibitors (SSRIs) and selective serotonin and noradrenaline inhibitors (SNRIs) are among the most commonly used drugs in the world in the pharmacotherapy of recurrent depressive disorders. Antidepressants affecting neural stem cells (NSCs) are believed to include selective noradrenaline reuptake inhibitors, monoamine oxidase inhibitors, and serotonin reuptake inhibitors [50,51]. Studies confirming their involvement in neurogenesis have shown increased levels of NSC proliferation with long-term treatment with fluoxetine, tranylcypromine, and reboxetine. During treatment with these drugs, the number of new neurons increased by 20–40% [52]. The unappreciated anti-inflammatory and anti-oxidant actions may constitute a potential mechanism of action of these agents [13]. Despite the large number of records displayed after entering the search terms, only a few met the inclusion criteria for this review. Most of the published studies were conducted in a small group of subjects, or their duration was too short to be included in the present review. Based on the reviewed studies, we conclude that the duration of treatment with the antidepressant drug in question and the time from treatment initiation to the measurement of the cytokine of interest significantly affected its level. Some of the studies included in the review revealed various levels of the inflammatory cytokines depending on the factor in question, the time points at which the levels of the inflammatory cytokines were measured, and the antidepressant drug used [21,22,23,41]. Equally ambiguous are the data on the prediction of treatment responses depending on baseline levels of the cytokines [19,23,27,28,31,41,43].

The effects of antidepressant drugs on IFN-γ varied. Increased IFN-γ levels were observed after treatment with duloxetine and paroxetine in patients with MDD [19,23], while decreases in IFN-γ concentrations to levels observed in healthy individuals were reported with treatment of sertraline, ketamine, and venlafaxine [19,22]. In the study by Chen et al., a significant decrease in IFN-γ was observed after 8 weeks of treatment with venlafaxine, but after the same duration of treatment with paroxetine, IFN-γ levels in patients with MDD increased [19]. In the 6-week study conducted in patients treated with sertraline, IFN-γ levels decreased already 6 weeks, with the decrease being independent of the treatment response [22]. No significant differences in the absolute values of cytokine levels at Week 6 were, however, reported during treatment with duloxetine [23].

Data on the effects of antidepressant drugs on CRP are equivocal. Effects of venlafaxine, fluoxetine, and paroxetine on CRP have been shown in studies by Chang et al. [32] and by Carboni et al. [27]. The meta-analysis did not show any effect of antidepressant drugs on CRP levels [25], and one study included in the review failed to show any effects of SSRIs on CRP levels [36].

Studies assessing the effects of antidepressant drugs on interleukin levels provided equivocal results. Lucassen et al. showed no direct effect of antidepressant drugs on the levels of IL-6, IL-1β, IL-1α, and TNF [53]. As regards interleukin-1, the study by Chen et al. [19] showed a decrease in IL-1β levels in the group of patients with MDD treated with venlafaxine and paroxetine. Those levels were similar to those observed in healthy individuals. Similar reduction in IL-1β concentrations to levels observed in healthy individuals after treatment of MDD with fluoxetine, paroxetine, and sertraline was reported in the study by Hernandez et al. [21]. In the study by Pérez-Sánchez et al. (2018), the Mann–Whitney U test revealed significant differences in the levels of IL-1β, and after 8 weeks of treatment, IL-1β returned to baseline [41]. In the study by Piletz et al., IL-1β levels after 8 weeks of treatment with venlafaxine increased [40]. ANOVA showed that fluoxetine was a significant predictor of the change in IL-1β levels [41] (Pérez-Sánchez et al., 2018). There are, however, studies that have not demonstrated any significant differences in IL-1β levels before and after treatment with fluoxetine [29]. Fornaro et al. (2013) distinguished a group of subjects who reacted early (after 6 weeks) to duloxetine and at the same time had a higher level of IL-1b compared with patients who did not improve treatment [23]. Data on the effects of antidepressant drugs on IL-1 are equivocal and further studies are required. The effects of various antidepressant drugs on the levels of the markers of interest are summarized in Table 2.

## 5. Conclusions

The analysis of the inflammatory factors confirms the numerous reports of their effect on the etiology and pathogenesis of depression. Of interest is the analysis of the effects of the individual antidepressant drugs, taking into account their effects on the various inflammatory markers. As it turns out, the effects of the drugs are equivocal, with individual drugs from the same class being able to affect factor levels in a different way. One might therefore ask whether taking the effects of individual antidepressant drugs into account could be of significance for the treatment response. Another question is whether it would be justified to select a drug based on the presentation of depression and comorbidities—e.g., an anti-inflammatory or neurotrophic agent? It is also justified to ask whether the factors included in the present review can predict treatment response. In conclusion, there is insufficient data to answer these questions. Most of the studies were conducted in a very small group of subjects, or the measurements were taken at one time point only or at insufficiently long intervals of time to be able to assess the outcomes of treatment and the effects of the treatment on the factors in question. Evidence related to the effects of antidepressant treatment on cytokine levels is still insufficient to propose a treatment regimen based on the immune model of depression. These findings suggest differential effects of these drugs on inflammatory factors. The effects of antidepressant drugs on inflammatory cytokines vary depending on the duration of treatment and the achieved treatment effect. Studies in a larger sample of patients are needed [19] (Chen et al., 2017). It is therefore necessary to conduct studies with larger numbers of patients.

## Figures and Tables

**Table 1:** The effects of antidepressant drugs on peripheral biomarkers.

Study	Publication Year	Number of Patients	Duration of the Study	Markers	Medications	Influence of Drugs on Markers	Results
Fornaro et al. [23]	2013	*N* = 30	6 weeks, 12 weeks	IL-1b, IL-2, IL-4, IL-10, IL-12, IFN-g, TNF-α	duloxetine	NO	6 weeks of treatment: no significant variations in mean cytokine plasma values; in early responders (ER) TNF-α levels decreased; in non-responders (ENR) IL-10 values decreased and IL-1b, IL-12, and IFN-g levels increased. Compared with controls, ER showed lower levels of IFN-g and TNF-α while ENR showed lower levels of IFN-g and IL-1b. 12 weeks: ENR—lower levels of IL-1b, IFN-g, and TNF-α compared with controls.
Wang et al. [24]	2019	*N* = 123	meta-analysis	IL-1β, IL-2, IL-4, IL-6, IL-10, TNF-α, and IFN-γ	escitalopram, citalopram, sertraline, fluoxetine, fluvoxamine, paroxetine	NO	Non-significant treatment effect.
Więdłocha et al. [25]	2017	*N* = 154	meta-analysis	IL-1ß, IL-2, IL-5, IL-6, IL-8, IL-10, CRP, TNF-α, IFN-γ	escitalopram, citalopram, sertraline, fluoxetine, fluvoxamine, venlafaxine, duloxetine, paroxetine, amitriptiline, clomipramine, nortriptiline, mirtazapine	NO	There was no statistically significant effect of antidepressant treatment on cytokines levels.
Köhler et al. [26]	2017	*N* = 242	meta-analisys	IL-6, TNF-α, IL-1b, IL-10, IL-4, IFN-y, IL-2, IL-8, CCL-2, CCL-3, IL-13, IL-17, IL-5, IL-7, IL-1R, sIL-2 receptor	escitalopram, citalopram, sertraline, fluoxetine, fluvoxamine, paroxetine, venlafaxine, duloxetine	NO	Levels of cytokine/chemokines were not significantly altered after antidepressant drug treatment.
Chen et al. [19]	2018	91 were completers for 8-week paroxetine (*n* = 50) or 8-week venlafaxine treatment (*n* = 41)	8 weeks	IFN-γ, TNF-α, IL-1β, IL-2, IL-4, IL5, IL-6, IL-8, IL-10, and GM-CSF	venlafaxine, paroxetine	IFN-y YES Venlafaxine: IL-5, IL-8 YES; IL-1 NO Paroxetine: IL-4 NO	8 weeks of treatment: venlafaxine—the mean IFN-γ, TNF-α, IL-4, IL-5, and IL-8 levels were significantly lower than in the paroxetine-treated group. Levels of the Th1 cytokines, IFN-γ, and TNF-α decreased after venlafaxine treatment, whereas IFN-γ and TNF-α increased after paroxetine treatment. Significant differences between paroxetine and venlafaxine treatment were observed in the change of cytokine levels such as IFN-γ, TNF-α, IL-4, IL-5, IL-8, and GM-CSF. After the 8-week paroxetine treatment, the mean IFN-γ and TNF-α levels increased in the ENR. IL-6 levels increased more in the ENR than in the ER. In the venlafaxine-treated group, the mean changes in cytokine levels did not differ significantly between ENR and ER. For levels of the Th2 and other cytokines, venlafaxine treatment caused a greater decrease in IL-4, IL-5, and IL-8 levels than did paroxetine treatment. After the 8-week venlafaxine treatment, the mean IL-1β and IL-8 levels had decreased and did not differ significantly from those of the healthy controls. The levels of IFN-γ and TNF-α increased after paroxetine treatment.
Dahl et al. [20]	2014	*N* = 50	12 weeks	IL-1b, IL-1Ra, IL-2, IL-5, IL-6, IL-7, IL-8, IL-10, IL-15, G-CSF, MIP-1a, TNF-α, (IFNg)	sertraline, escitalopram, citalopram, venlafaxine, mirtazapine, benzodiazepines, lamotrigine, psychotherapy	YES	Seven of the nine cytokines that were elevated at baseline were significantly reduced after the 12 weeks of therapy compared with baseline: IL-1Ra, IL-6, IL-7, IL-8, IL-10, G-CSF, and IFNg. The levels of IL-1b and IL-5 were not significantly reduced.
Brunoni et al. [22]	2013	*N* = 18	6 weeks	IL-2, IL-4, IL-6, IL-10, IL-17A, IFN-γ, TNF-α	sertraline	YES/no	The plasma levels of all cytokines (except TNF-α) decreased during the treatment. No significant results were found comparing cytokine plasma levels at baseline according to clinical response.
Hernandez et al. [21]	2013	*N* = 31	52 weeks	IL-1*β*, IL-2, IFN-*γ*, IL-4, IL-10, and IL-13	fluoxetine, setraline, paroxetine	YES	IFN-γ levels fluctuated during the treatments and showed significant changes. Before treatment, patients had lower IFN-γ levels. At the end of treatment, IFN-γ levels were comparable with those of healthy volunteers.
Carboni et al. [27]	2019	*N* = 103	10	IL-6, IL- 10, TNF-α, TNFRII, BDNF, CRP, MMP9, and PAI1	venlafaxine (51), paroxetine (52)	YES	Paroxetine: increase in biomarker levels after treatment correlated with reduction in depression symptomatology for TNF-α, IL-6, IL-10, and CRP. Responders showed higher baseline IL-10 levels compared with non-responders. Venlafaxine—significant association between baseline levels of CRP and changes in HAM-D in males.
Manoharan et al. [28]	2016	*N* = 73 MDD patients (39 responders and 34 non-responders)	6	IL-6	fluoxetine	NO	IL-6 levels were significantly higher in MDD patients when compared with controls. Pre-treatment serum IL-6 concentrations did not significantly differ between responders and nonresponders. Both groups also did not show significant reduction in the IL-6 levels post-treatment. A significant correlation was observed between the percentage change in IL-6 and percentage change in depression score in responders.
Jazayeri et al. [29]	2010	*N* = 14	8	IL-1, IL-6	fluoxetine	NO	Serum concentrations of IL-1β and IL-6 did not change significantly after intervention based on repeated-measures ANOVA.
Halaris et al. [30]	2015	*N* = 20	8 and 12 weeks	CRP, TNF-α, IL-1, IL-4, IL-6, IL-8, IL-10	escitalopram	NO	Closer inspection of the averages indicates a tendency for some biomarkers to decline at week 8 but to bounce back up again at week 12 (e.g., IL-6, hsCRP, TNF-α). At 8 weeks of ESC monotherapy, when the HAM-D and related scores improved significantly, hsCRP, TNF-α, IL-6, IL-8, IL-10, and MCP1 trended lower.
Eller, et al. [31]	2008	*N* = 100 74 responders 45 HV	4 and 12 weeks	IL-8, TNF-α	escitalopram	TNF—YES, The rest NO	The comparison of baseline cytokine levels between responders, non-responders, and healthy subjects demonstrated statistically significant between-groups difference for TNF-α but not for other cytokines. Escitalopram: the concentration of TNF-α did not significantly change during 12 weeks of treatment; however, it increased to normal levels in the group of responders. Week 12, ANCOVA did not show differences in cytokine levels between treatment groups and healthy volunteers.
Chang et al. [32]	2020	*N* = 149	6	CRP	venlafaxine (*n* = 76) fluoxetine (*n* = 73)	NO	The baseline CRP levels were significantly correlated with treatment response at week 2. Patients with higher CRP levels had a poorer treatment response. CRP level had increased significantly after six weeks of treatment in patients receiving either antidepressant. The CRP level remained significantly high after six weeks of treatment in patients with a higher baseline level.
Yang et al. [33]	2019	review					Raison et al. (2013) [7]—baseline values of hsCRP > 5 mg/L predicted a greater decrease in HAMD-17 scores in infliximab-treated patients than placebo-treated subjects. Papakostas et al. (2014) [34]—changes on the HAMD28 scoring list were significantly greater than baseline scores in a subpopulation with hsCRP levels above the study median value when comparing L-methylfolate with placebo treatment. Kruse et al. (2018) [35]—baseline CRP was not associated with changes of MADRS scores, but it was significantly correlated with the end-of-treatment scores for women. Chen et al. (2018) [19]—log-transformed CRP levels were not predictive for ketamine treatment response.
Tuglu et al. [36]	2003	*N* = 26	6	TNF-α, CRP	citalopram, sertraline, fluoxetine, fluvoxamine, paroxetine	YES	CRP levels after treatment were significantly lower than those on admission. Comparison of pre- and post-treatment measurements revealed that TNF-α, CRP, and leukocyte count decreased to levels comparable with those of the control subjects.

**Table 2:** The effects of antidepressant drugs on various cytokines.

Study	Medication	Effect *
	IL-1	
Chen et al. [19]	venlafaxine	−
	paroxetine	−
Piletz et al. [40]	venlafaxine	+
Jazayeri et al. [29]	fluoxetine	N/C
Hernandez et al. [21]	SSRIs (fluoxetine, paroxetine, sertraline)	+
Fornaro et al. [23]	duloxetine	+/−, +(non responders)
Halaris et al. [30]	escitalopram	N/C
	**IL-6**	
Carboni et al. [27]	venlafaxine	N/C
	paroxetine	+
Pérez–Sánchez et al. [41]	fluoxetine	− after 6 weeks, +/− after 8 weeks
Manoharanet et al. [36]	fluoxetine	N/C
Yoshimura et al. [43]	Sertraline	− (responders)
	paroxetine	− (responders)
Jazayeri et al. [29]	fluoxetine	N/C
Halaris et al. [30]	escitalopram	8 week − ; later N/C
	**IL-8**	
Chen et al. [19]	venlafaxine	−
	paroxetine	−
Eller et al. [31]	escitalopram	N/C
Halaris et al. [30]	escitalopram	N/C
	**IL-10**	
Carboni et al. [27]	venlafaxine	N/C
	paroxetine	+/−, + (responders)
Fornaro et al. [23]	duloxetine	+/−, − (non responders)
Halaris et al. [30]	escitalopram	N/C
Chen et.al. [19]	venlafaxine	−
	paroxetine	N/C
	**TNF**	
Eller et al. [31]	escitalopram	N/C
Carboni et al. [27]	venlafaxine	N/C
	paroxetine	+ u responders
Pérez–Sánchez et al. [41]	fluoxetine	+/−
Fornaro et al. [23]	duloxetine	+/−, − u responders vs. nr
Halaris et al. [30]	escitalopram	8 week − ; 12 week N/C
Chen et.al. [19]	venlafaxine	−
	**IFN-y**	
Fornaro et al. [23]	duloxetine	+
Chen et al. [19]	paroxetine	+
	venlafaxine	−
Brunoni et al. [22]	sertraline	−
	**CRP**	
Chang et al. [32]	venlafaxine	+
	fluoxetine	+
Carboni et al. [27]	paroxetine	+
Tugku et al. [36]	SSRI (citalopram, sertraline, fluoxetine, fluvoxamine, paroxetine)	−
Więdłocha et al. [25]	SSRI (escitalopram, citalopram, sertraline, fluoxetine, fluvoxamine, paroxetine)	N/C
	SNRI (venlafaxine, duloxetine)	N/C
	TCA (amitriptiline, clomipramine, nortriptiline)	N/C

* + increase, − decrease, N/C unchanged.

## Data Availability

Not applicable.
